# Transcriptomic Analysis of Acclimation to Temperature and Light Stress in *Saccharina latissima* (Phaeophyceae)

**DOI:** 10.1371/journal.pone.0044342

**Published:** 2012-08-28

**Authors:** Sandra Heinrich, Klaus Valentin, Stephan Frickenhaus, Uwe John, Christian Wiencke

**Affiliations:** 1 Department of Functional Ecology, Alfred Wegener Institute for Polar and Marine Research, Bremerhaven, Germany; 2 Department of Polar Biological Oceanography, Alfred Wegener Institute for Polar and Marine Research, Bremerhaven, Germany; 3 Department of Biotechnology, Hochschule Bremerhaven, Bremerhaven, Germany; 4 Department of Ecological Chemistry, Alfred Wegener Institute for Polar and Marine Research, Bremerhaven, Germany; 5 Department of Functional Ecology, Alfred Wegener Institute for Polar and Marine Research, Bremerhaven, Germany; Rutgers University, United States of America

## Abstract

Kelps, brown algae of the order *Laminariales*, dominate rocky shores and form huge kelp beds which provide habitat and nurseries for various marine organisms. Whereas the basic physiological and ecophysiological characteristics of kelps are well studied, the molecular processes underlying acclimation to different light and temperature conditions are still poorly understood. Therefore we investigated the molecular mechanisms underlying the physiological acclimation to light and temperature stress. Sporophytes of *S. latissima* were exposed to combinations of light intensities and temperatures, and microarray hybridizations were performed to determine changes in gene expression patterns. This first large-scale transcriptomic study of a kelp species shows that *S. latissim*a responds to temperature and light stress with a multitude of transcriptional changes: up to 32% of genes showed an altered expression after the exposure experiments. High temperature had stronger effects on gene expression in *S. latissima* than low temperature, reflected by the higher number of temperature-responsive genes. We gained insights into underlying molecular processes of acclimation, which includes adjustment of the primary metabolism as well as induction of several ROS scavengers and a sophisticated regulation of Hsps. We show that *S. latissima,* as a cold adapted species, must make stronger efforts for acclimating to high than to low temperatures. The strongest response was caused by the combination of high temperatures with high light intensities, which proved most harmful for the alga.

## Introduction

Marine macroalgae are key components of coastal ecosystems and play an important role as the nutritional basis in marine communities [1]–[3]. Although they cover only a small percentage of the area of the world's ocean, they account for up to 10% of the global oceanic primary production [4], [5]. The largest biogenic structures found in benthic marine systems are kelp beds which mostly consist of macroalgae from the order Laminariales [3]. Kelp beds dominate rocky coastal shores of the world's cold-water marine habitats; they provide a unique three-dimensional habitat for marine organisms by offering a physical structure for shelter, protection from predators and nurseries ground as well as food for various marine animals [2], [6]. Kelps are cold-temperate water species; their high abundance and biomass in sub-polar and cold-temperate regions suggests that they are well adapted to low light and low temperatures [1], [7], [8]. Cold adapted species developed mechanisms, such as changes in gene expression to maintain sufficient rates of enzyme-catalyzed reactions and modifications within the thylakoid membrane system, affecting photosynthetic electron transport, to overcome the constraints of exposure to low temperature [9], [10].

Primary factors responsible for the vertical zonation and geographical distribution of kelps are light, including UV radiation, temperature and the availability of nutrients, the latter sometimes correlated with lower temperatures in upwelling regions [11]–[14]. Current ocean temperature rises caused by global warming and stratospheric ozone depletion thus likely will influence zonation, distribution patterns, and the performance of kelp in their present habitats. The perennial kelp *Saccharina latissima* ( =  formerly *Laminaria saccharina*) is a common species in polar to temperate coastal waters and is distributed circumpolar in the northern hemisphere [15], [16]. The natural growth sites of *S. latissima* are clear and turbid coastal waters, it occurs from the intertidal down to 30 m depth; as a result *S. latissima* is exposed to a wide range of temperature and light conditions [17]. Both the wide latitudinal and vertical distribution of this species might be correlated to ecotypic differentiation of populations with respect to light and temperature. Ecotypic differentiation was reported for sporophytes and gametophytes of *S. latissima* from Long Island Sound (USA) and the Atlantic coast from Maine (USA) [17]–[19] as well as for gametophytes from Spitsbergen (Norway) and Helgoland (Germany) [20]; the variation in light- and temperature related traits is suggested to have a genetic basis.

The effects of the abiotic factors light and temperature on the physiology and growth of *S. latissima* have been studied extensively [7], [21]–[24], yet the molecular processes of acclimation and adaption are still poorly understood. Gene expression data for kelps are scarce, until now only three cDNA approaches have been published. Crépineau et al. 2000 characterized 905 expressed sequence tags (ESTs) of life cycle stages of *Laminaria digitata* [25], Roeder et al. 2005 established a cDNA library of 1985 ESTs from *Laminaria digitata* protoplasts [26]. More recently a subtractive cDNA approach on oligoguluonate-induced transcriptional defense responses was conducted [27]. For sessile organisms such as kelps acclimation to environmental changes in order to maintain cellular function is particularly important. In sessile plants for example extrinsic stress resulting from changes in abiotic factors, e.g. light and temperature, is regarded as the most important stress agent [28]. Excessive light causes photo-oxidative stress through an over-reduction of the photosynthetic electron transport chain; electrons from the light reaction are transferred to oxygen, which leads to the formation of reactive oxygen species (ROS), such as superoxide radicals and hydrogen peroxides [29]–[31]. Low temperature alter significantly plant metabolism, its physiology, and plant productivity, given that in general enzyme activity decreases with declining temperatures [32], [33]. Reduced activity of the Calvin cycle results in a decreased production of the final electron acceptor NADP^+^, which may lead to electron transfer from reduced ferredoxin to oxygen and therefore to the formation of ROS [34]. Heat stress on the one hand leads to the degradation and dysfunction of proteins, on the other hand to an uncoupling of pathways, resulting in formation of ROS, which – in turn – induces lipid peroxidation [35]–[38]. Although the abiotic factors light and temperature operate through different mechanisms, both lead to the formation of ROS. ROS cause cellular damage in terms of denaturation of nucleic acids, proteins, polysaccharides and lipids [39]–[41]. Cells are able to counteract ROS with a sophisticated network of non-enzymatic and enzymatic systems, which scavenge the various ROS intermediates [42]–[44]. The non-enzymatic antioxidants include the major cellular redox buffers ascorbate (Asa) and glutathione (GSH) [45], [46]. Enzymatic ROS detoxification includes the conversion of superoxide radicals into hydrogen peroxide and oxygen by superoxide dismutase (SOD), subsequently hydrogen peroxide is eliminated in the ascorbate–glutathione cycle by ascorbate peroxidase (APX) and glutathione reductase (GR) [44], [47], [48]. However, ROS are not only potentially harmful, but also part of a subtle network of signaling reactions [49]. ROS as well as the redox state of several regulatory redox-reactive key molecules, such as thioredoxin and glutathione, are signals which regulate expression e.g. of photosynthesis-related genes [34], [42].

This study aims to investigate the molecular mechanisms underlying physiological acclimation to temperature and light stress in *S. latissima* from the Arctic (Spitsbergen). In order to analyze the sensitivity of this cold adapted species to either high or low temperatures in combination with high light, sporophytes were exposed for 24 h to 5 different combinations of light intensities and temperatures. Photosynthetic efficiency, measured as variable fluorescence of PS II, was determined before and at the end of the experiment. Changes in gene expression levels were assessed through oligonucleotide microarrays. We expected that *S. latissima*, as a cold adapted species, would be more negatively effected by high than low temperatures and that this will be reflected in the photosynthetic fitness and the changing gene expression profiles of the organism.

## Results

### Photosynthetic efficiency

**Figure 1:**
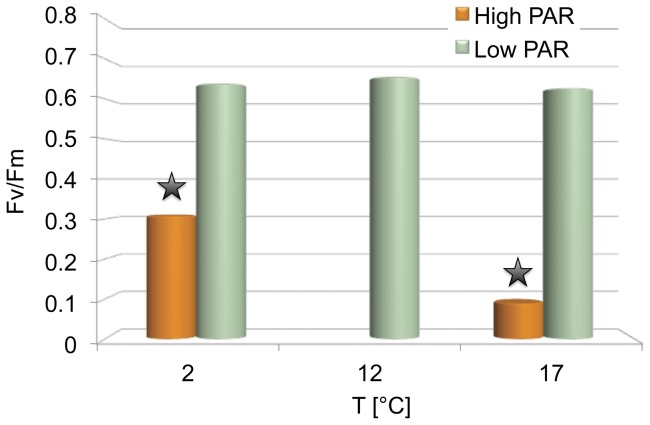
Efficiency of PS II (*Fv/Fm*) after 24 h exposure to different temperature and radiation conditions. The figure displays the maximum quantum yield of PS II in *S. latissima* after 24 h of exposure to different temperatures (2°C/17°C) and different light conditions (low/ high PAR). Standard deviations are represented by vertical bars (n = 5). Asterisk mark significant differences in efficiency of PS II (two-way ANOVA with repeated measurements, n = 5, p<0.01; post hoc Tukey test HSD, p<0.01).

Initial mean maximum quantum yield of PS II (*F*v/*F*m) was 0.633±0.02. Maximum quantum yield of PS II remained stable at low photosynthetically active radiation (PAR) under the three tested temperature regimes (control low PAR 12°C; *F*v/*F*m  = 0.653±0.01). A significant decrease (p<0.01) in efficiency of PS II was observed in sporophytes exposed to high photosynthetically active radiation at 2°C (*F*v/*F*m  = 0.308±0.07) and 17°C (*F*v/*F*m  = 0.09±0.003). Strongest photoinhibition occurred under high photosynthetically active radiation and a temperature of 17°C (Figure 1).

### Gene expression patterns in response to light and temperature stress

**Figure 2:**
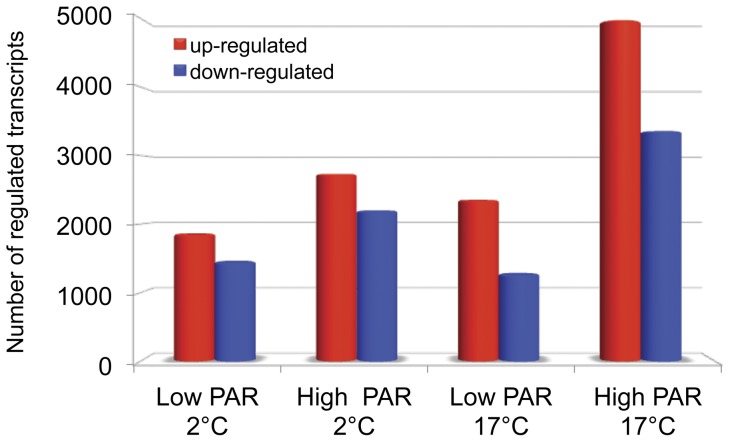
Numbers of differentially up- (red bars) and down-regulated (blue bars) genes after various stress treatments. The figure displays the numbers of up- and down regulated genes in *S. latissima* after 24 h of exposure to different temperatures (2°C/17°C) and different light conditions (low/high PAR). Identification of regulated ESTs is based on microarray hybridizations and evaluated with an ANOVA against a control treatment with n = 4 and p<0.01, followed by a post hoc Tukey test (HSD, p<0.01).

Oligonucleotide microarrays covering 26,224 transcripts were used to determine changes in gene expression patterns at 3 temperatures (2°C/12°C/17°C) and high photosynthetically active radiation (PAR) stress. Of these 10,915 transcripts (42%) showed different expression patterns under at least one stress treatment compared to the control treatment (12°C & low PAR). The strongest effect on gene expression was observed in the high PAR/17°C treatment when 8,334 genes (32%) were affected. The remaining three treatments (2°C & high/low PAR, 17°C low PAR) caused transcriptional changes for 13–19% (3,289–4,920) of the genes (Figure 2).

### Identification of temperature- and light-regulated genes

**Figure 3:**
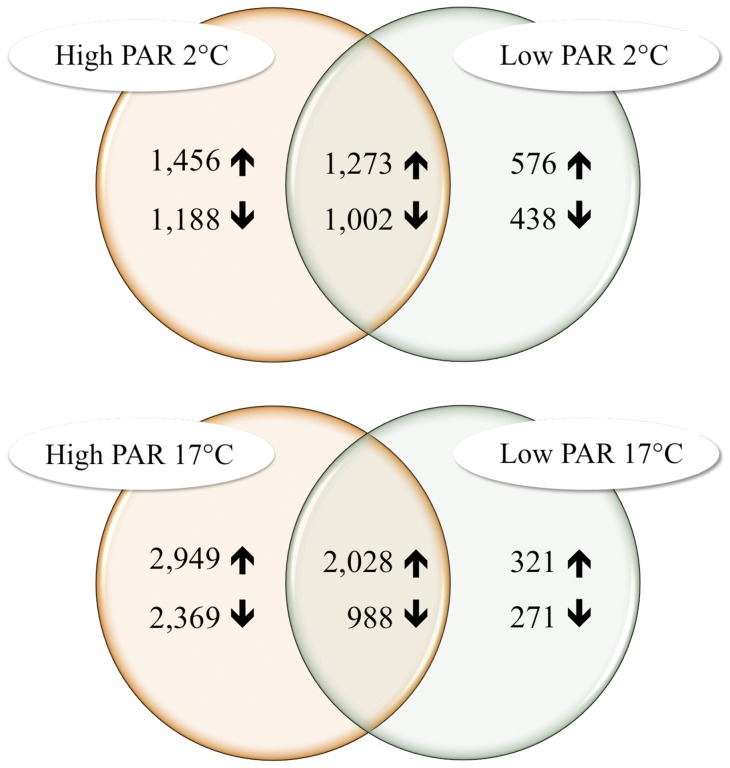
Venn diagram of responsive ESTs after 24 h exposure to different light and temperature conditions. Numbers of responsive ESTs in *S. latissima* after exposure to different experimental conditions. Regulated transcripts are seperated in up (↑) and down (↓) regulated ESTs. The intersections display the number of ESTs regulated in both treatments.

A cross comparison was performed to identify an overlap of ESTs responsive to either high or low temperature within the different light treatments (Figure 3).

High temperature was found to up-regulate the expression levels of 2,028 genes and down-regulate those of 988. The effect of low temperature was less pronounced in that 1,273 genes were found to be up-regulated and 1,002 genes down-regulated. The cross comparison revealed that the amount of high PAR responsive ESTs is dependent on temperature: at high PAR and low temperature 1,456 genes were induced whereas 1,188 genes were repressed; at high PAR and high temperature 2,949 genes were induced and 2,369 genes repressed.

Gene enrichments were performed to assign biological processes to these large groups of responsive ESTs. We analyzed whether the abundance of certain Gene Ontology (GO) terms within the GO root category biological process is significantly different between the groups of the cross comparison versus the whole gene set on the microarray. Special emphasis was given to the ratio of biosynthetic versus catabolic processes at different treatments. In the group of genes simultaneously up-regulated under both light conditions at low temperature the GO term one-carbon metabolic process (GO:0006730) was over-represented. The high PAR low temperature up-regulated genes revealed 52 enriched GO terms of the roots biological process (for a detailed list see Table S1). Among these more catabolism-related GO terms were detected than GO terms related to biosynthesis. Among the down-regulated genes at low temperature as well as at high PAR and low temperature no over-represented GO terms could be found. The set of down-regulated high temperature-responsive ESTs featured 29 over-represented GO categories, often representing catabolic as well as biosynthetic processes of a metabolic process, e.g. monosaccharide biosynthetic process (GO:0046364) and monosaccharide catabolic process (GO:0046365). The highest abundance of enriched GO terms was observed among the high PAR high temperature down-regulated genes. We detected more catabolism-related GO terms then biosynthesis-related GO terms. 125 over-represented GO terms were identified, including several crucial metabolism categories e.g. photosynthesis (GO:0015979), carbohydrate metabolic process (GO:0005975), and cellular amino acid metabolic process (GO:0006520).

### Gene enrichments of single treatment conditions

Gene enrichments were performed for all significantly regulated genes of the different stress conditions. The analyses showed that temperature stress combined with high PAR stress led to a stronger shift in gene expression compared to the control treatment than temperature stress alone. We detected 50 over-represented GO terms among the regulated genes under low temperature and high PAR and 89 enriched GO terms within the high temperature and high PAR regulated genes, but only 5 respectively 4 GO terms within the regulated genes under low and high temperature stress alone. For a detailed list see Table S2.

The high PAR treatments showed 23 commonly enriched GO terms, e.g. plastid (GO:0009536), and cellular component organization (GO:0016043). The high PAR treatment at low temperature additionally resulted in a higher abundance of GO terms correlated to amino acid metabolism such as cellular amino acid biosynthetic process (GO:0008652), and cellular amino acid and derivative metabolic process (GO:0006519). High PAR treatment at high temperature on the contrary led to an enhanced appearance of GO terms associated with photosynthetic components, catabolism, and biosynthetic processes, in particular cellular nitrogen compound biosynthetic process (GO:0044271) and porphyrin biosynthetic process (GO:0006779). We found no shared enriched GO terms within the low PAR treatments.

### Summary of transcriptional changes based on KOGs

**Figure 4:**
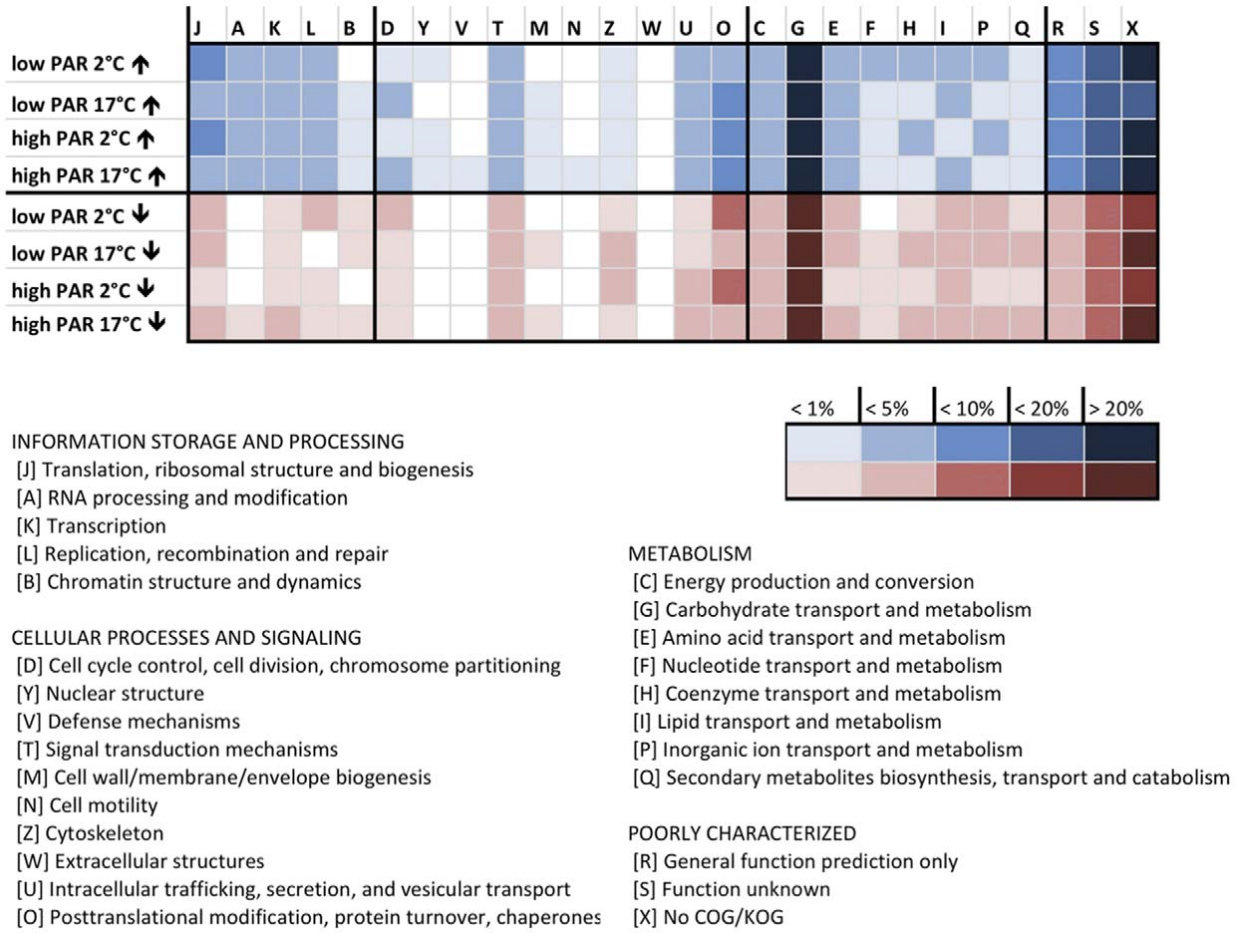
KOG category distributions of differentially expressed transcripts determined by microarray hybridizations. The figure displays the KOG category distribution of up (↑) and down (↓) regulated transcripts in *S. latissim*a after exposure to the stress treatments in comparison to the control treatment. Color intensities reflect the amount of ESTs per group calculated in percent of total ESTs grouped into KOGs with known or general function prediction.

Distribution of differentially regulated transcripts from the different stress treatments based on KOG categories indicate a broad response concerning multiple cellular functions (Figure 4). A temperature of 2°C for example yielded in stronger induction of transcripts of the categories ‘translation, ribosomal structure and biogenesis’ and ‘inorganic ion transport and metabolism’, furthermore to stronger repression of genes associated with the category ‘posttranslational modification, protein turnover, chaperones’. A temperature of 17°C caused induction of a higher number of genes correlated to the category ‘cell cycle control, cell division, chromosome partitioning’ as well as stronger repression of transcripts of the categories ‘coenzyme transport and metabolism’, ‘secondary metabolites biosynthesis, transport and catabolism’ and ‘cell wall/membrane/envelope biogenesis’.

### Classification of over-represented KEGG pathways

KOBAS analyses resulted in the identification of several significantly enriched pathways within the different treatments (Table 1). At low temperature under both light conditions up-regulated genes involved in glycine, serine, and threonine metabolism were affected. Among the up-regulated genes under low temperature and high PAR we observed additionally an increased regulation of alanine, aspartate, and glutamate metabolism, and aminoacyl-tRNA biosynthesis. Common features at high temperature included down-regulation of transcripts involved in carbon fixation. Furthermore the high PAR and high temperature condition led to a significant down-regulation of several metabolic pathways such as glycolysis/gluconeogenesis, glutamate, fructose and mannose, nitrogen, and porphyrin and chlorophyll metabolism as well as collecting duct acid secretion. For the latter genes contributing to this pathway are mostly encoding for different isoforms of the V-type proton ATPase. A full list of genes contributing to the results of the KOBAS analysis can be retrieved from the supplemental material (Table S3).

**Table 1:** Over-represented KEGG pathways among the significantly up- and down-regulated genes under different stress conditions.

Treatment	Regulation	KEGG pathway	KO Id
***2°C high PAR/2°C low PAR***	***up***	Glycine, serine and threonine metabolism	ko00260
***2°C high PAR***	***up***	Alanine, aspartate and glutamate metabolism	ko00250
		Aminoacyl-tRNA biosynthesis	ko00970
***17°C high PAR/17°C low PAR***	***down***	Carbon fixation in photosynthetic organisms	ko00710
***17°C high PAR***	***down***	Collecting duct acid secretion	ko04966
		Glycolysis/Gluconeogenesis	ko00010
		Fructose and mannose metabolism	ko00051
		Nitrogen metabolism	ko00910
		Porphyrin and chlorophyll metabolism	ko00860

Enriched KEGG pathways were identified with a hypergeometric test (p<0.01).

### Manual classification of genes responsive to temperature and oxidative stress

To investigate the mechanisms involved in the response to temperature and high PAR mediated oxidative stress, as well as the effects from these stressors on the state of photosynthetic components, we examined manually transcriptional changes of genes encoding photosynthetic components (Table 2), ROS scavengers (Table 3), heat shock proteins (Hsps) (Table 4), and transcripts involved in proteolysis (Table 5). A full list of the regulated genes can be retrieved from the supplemental material (Table S4). We observed a strong down-regulation of transcription for most of the photosynthetic components in response to the high PAR 17°C treatment, the strongest transcriptional changes of up to 61.7 fold occurred in genes correlated to the light harvesting complex (contig15369, contig07943, contig08792). Additionally transcripts encoding the photosystem II (e.g. photosystem II 12 kDa extrinsic protein, photosystem II cp47 chlorophyll apoprotein, and photosystem II D2 protein), and transcripts of thylakoid luminal proteins (contig07691, contig24607) were significantly repressed. The 17°C low PAR treatment resulted in significantly down-regulated transcripts coding for the fucoxanthin chl a c lhca clade, light harvesting complex protein 4, light-harvest protein, and photosystem I reaction center subunit XI, for them transcriptional changes between 2.1–2.7 fold were observed. Low temperature treatments on the contrary led to a significant induction of photosynthetic component transcripts: some transcripts showed a similar abundance within the high and low light treatment, e.g. light harvesting complex protein and photosystem II protein Y. Other up-regulated transcripts at low temperature such as photosystem II biogenesis protein psp29 and thylakoid lumenal protein exhibited higher transcriptional changes within the high PAR treatment compared to the low PAR treatment.

**Table 2:** Differential regulated genes encoding photosynthetic components.

Contig name	Putative gene product	Annotation e-Value	Fold change			
			2°C low PAR	2°C high PAR	17°C low PAR	17°C high PAR
Contig12790	Cytochrome b6-f complex	1.9e-55	−1.1	−1.2	−1.3	**−4.5**
Contig06404	Fucoxanthin-chlorophyll a-c binding protein	1.6e-82	1.1	−1.1	−1.6	**−8.6**
Contig15369	Fucoxanthin chl a c lhca clade	6.9e-59	−1	−1.8	**−2.1**	**−61.7**
Contig12435	Light harvesting complex protein	1.2e-52	**2.1**	**2.9**	1.1	1.2
Contig07943	Light harvesting complex protein 4	5.6e-66	−1.1	−1.5	**−2.6**	**−49.7**
Contig08792	Light-harvest protein	6.4eE-85	−1.5	−1.7	**−2.7**	**−16.5**
Contig00811	Photosystem I reaction center subunit xi	8.9e-40	−1	1.2	**−2.2**	**−8.9**
Contig06470	Photosystem II 12 kDa extrinsic protein	2.4e-49	−1.1	−1.3	−1.5	**−8.9**
Contig02889	Photosystem II biogenesis protein psp29	2.1e-76	**2.8**	**5.4**	−1.1	−1.1
Contig01925	Photosystem II reaction centre protein D1/psba	0	1.7	1.5	1.2	**−3.7**
Contig02559	Photosystem II cp47 chlorophyll apoprotein	0	1.4	1.3	−1.9	**−20.9**
Contig03429	Photosystem II D2 protein	0	1.3	1.9	−1.3	**−14.4**
Contig14092	Photosystem II protein	7.5e-89	1.4	**2.6**	−1.7	**−2.9**
Contig13305	Photosystem II protein y	2.6e-07	**2.6**	**2.4**	1.9	−1.8
Contig28409	Photosystem II stability assembly factor	5.7e-73	1.9	**2.6**	−1.3	−1.8
Contig02889	Thylakoid protein	6.4e-14	**2.8**	**5.4**	−1	−1.1
Contig05910	Thylakoid luminal 17.4 kDa	1.1e-24	1.5	**2.2**	−1.4	−1.7
Contig07691	Thylakoid luminal 15 kDa protein	2.5e-36	**2.7**	**4.7**	−1.4	**−2.3**
Contig24607	Thylakoid luminal protein	1.1e-13	1.1	1.4	−1.7	**−3.1**

All displayed genes were differentially expressed with p<0.01 and a fold change >2 (numbers in bold).

**Table 3:** Differentially regulated genes encoding ROS scavenging enzymes.

Contig name	Putative gene product	Annotation e-Value	Fold change				
			2°C low PAR	2°C high PAR	17°C low PAR	17°C high PAR	
Contig09006	Chloroplastic alternative oxidase	9e-46	**2**	**3.1**	1.7	**3.9**	
Contig21020	Glutaredoxin	8.6e-09	−1.6	1.8	**2.4**	**2.8**	
Contig03562	Glutathione reductase	8.3e-112	**2.8**	**5.1**	1.4	**2.1**	
Contig03637	Glutathione S-transferase	1.3e-80	**−5.6**	**−2.8**	**5.2**	**9**	
Contig00051	L-ascorbate peroxidase	1.9e-112	1.8	**2.5**	1.5	1.2	
Contig05892	Superoxide dismutase [Fe]	1.3e-69	−1.2	−1.3	**2**	1.9	
Contig11011	Superoxide dismutase mitochondrial precursor	3.7e-88	−1	1.1	**3.2**	**5.2**	
Contig14359	Thioredoxin chloroplastic	8.4e-25	1.9	**2.6**	1.8	**2.9**	
Contig08467	Thioredoxin reductase chloroplastic	9.2e-36	**6.1**	**9.4**	1.9	**3.8**	

All displayed genes were differentially expressed p<0.01 and with a fold change >2 (numbers in bold).

**Table 4:** Differentially regulated genes encoding heat shock proteins.

Contig name	Putative gene product	Annotation e-Value	Fold change			
			2°C low PAR	2°C high PAR	17°C low PAR	17°C high PAR
Contig12085	Chaperone protein DnaJ	6.5e-56	**2.2**	**2.6**	1.9	**2.2**
Contig01237	Chaperone protein DnaK	5.8e-144	−1.3	**2.1**	**8.8**	**11.2**
Contig06341	Heat shock 70 kda protein 4	4.4e-34	1.2	1.4	1.6	**3**
Contig24742	Heat shock 70 kda protein 5	2.5e-37	1.9	**2.5**	**4.4**	**34.2**
Contig28226	Heat shock cognate 70 kda protein 1	4.9e-66	−1.1	1	1.8	**3.4**
Contig06811	Heat shock cognate 70 kda protein 3	3.6e-59	**2**	**2.1**	1.1	**2.4**
Contig13216	Heat shock factor-binding protein 1	1.5e-11	−1.1	−1.1	1.2	**3.4**
Contig10777	Heat shock protein 33 homolog	1.9e-33	**2.4**	**3.6**	−1.4	**−2.6**
Contig10085	Heat shock protein 60	7.7e-42	**2.5**	**3.7**	1.3	1.4
Contig09075	Heat shock protein 90	2.5e-145	**2**	**4**	1.9	1.8
Contig11643	Heat shock protein mitochondrial mthsp70	1.5e-07	−1.3	−1	1.5	**2.7**
Contig09074	Heat shock-like 85 kda protein	3.3e-09	1.2	**2.4**	**2**	**2**
Contig04604	Mthsp70-associated motor and chaperone protein	1.2e-07	1.1	1.5	**2.4**	**6**

All displayed genes were differentially expressed with p<0.01 and a fold change >2 (numbers in bold).

**Table 5:** Differentially regulated genes encoding proteins involved in proteolysis.

Contig name	Putative gene product	Annotation e-Value	Fold change			
			2°C low PAR	2°C high PAR	17°C low PAR	17°C high PAR
Contig07342	26s protease regulatory subunit 6a homolog	2e-156	−1.2	1.3	1.9	**3.3**
Contig11906	26s protease regulatory subunit 6b homolog	8.7e-174	−1	−1.3	1.6	**2.1**
Contig14531	26s protease regulatory subunit 8 a	1.6e-164	−1.1	−1.9	1.8	**2.1**
Contig21902	ATP-dependent clp protease proteolytic subunit	3.2e-36	1.3	**2.2**	1	1.5
Contig09850	ATP-dependent protease atpase subunit	8.9e-117	1.1	1.1	1.4	**4.7**
Contig02945	Cathepsin b-like cysteine proteinase 5	8.4e-31	−1	−1.1	−1.1	**3.1**
Contig17610	Cysteine proteinase 2	3.6e-12	−1.4	−1.4	1.3	**2.5**
Contig00569	Papain family cysteine protease containing protein	2.6e-56	−1.1	−1.2	**2.3**	**9.2**
Contig03402	Proteasome subunit alpha type 2	2.1e-119	−1.3	−1.3	**2.2**	**2.6**
Contig13292	Ubiquitin	1.1e-55	−1.1	1.5	1.2	**3.1**
Contig12782	Ubiquitin conjugation factor	7.7e-162	−1.3	−1.4	1.6	**2.4**
Contig07882	Ubiquitin specific protease 39 and scrap assembly	1.5e-112	1.1	−1.1	1.3	**2.6**
Contig13903	Ubiquitin-conjugating enzyme	2.5e-53	−1.4	−1.3	1.2	**4.4**
Contig02894	Ubiquitin-like modifier-activating enzyme 1	5.8e-119	1.5	−1.2	**2.1**	**4.3**

All displayed genes were differentially expressed with p<0.01 and a fold change >2 (numbers in bold).

We found a significant transcriptional regulation for several genes encoding antioxidative enzymes. Transcripts encoding the chloroplastic alternative oxidase, glutathione reductase, and thioredoxin reductase showed a significant up-regulation at all stress treatments except at low light/2°C. The highest transcriptional response for the chloroplastic alternative oxidase appeared under the high PAR 17°C treatment, whereas the induction of glutathione reductase and thioredoxin reductase was strongest at the low temperature/high PAR condition. Transcripts of glutaredoxin, glutathione S-transferase, and mitochondrial superoxide dismutase precursor were up-regulated at high temperatures and featured the highest transcript abundances at the high temperature/high PAR condition. Superoxide dismutase [Fe] in contrast was significantly up-regulated at low PAR 17°C, whereas transcripts coding the l-ascorbate peroxidase featured an induction at high PAR 2°C. Additionally we observed enhanced transcript abundance for chloroplastic thioredoxin at both high PAR conditions. The only significant down-regulation among transcripts correlated to ROS scavenging mechanism was observed for the glutathione S-transferase at the low temperature treatments.

We were able to identify several differently expressed Hsps genes; here we observed a diverse response of transcripts not only to high temperature, but also to low temperature. The strongest induction of Hsps occurred after the exposure to high PAR at 17°C with an 34.2 fold up-regulation for the heat shock 70 kDa protein 5, in addition we observed four chaperones, e.g. the heat shock 70 kDa protein 5 and the mitochondrial heat shock protein mtHSP70, exclusively up-regulated in response to this treatment. The transcript abundances of the chaperone protein DnaJ and the heat shock cognate 70 kDa protein 3 were significantly enhanced after all treatments except at the high temperature low PAR treatment, whereas chaperone protein DnaK, heat shock 70 kDa protein 5, and heat shock-like 85 kDa protein were up-regulated at all conditions except the low temperature low PAR condition, the heat shock 70 kDa protein 5 and the heat shock-like 85 kDa protein featured a stronger up-regulation at 17°C. Three Hsps, heat shock protein 33 homolog, heat shock protein 60, and heat shock protein 90 were significantly up-regulated in response to low temperature stress at both light conditions, but with a stronger induction at high PAR 2°C. Transcripts of the mthsp70-associated motor and chaperone protein exhibit a significantly higher abundance within the high temperature treatments with a stronger expression after the high PAR 2°C treatment.

We detected a significant induction of the 26s protease regulatory subunit 6a, 6b, and 8a at high PAR 17°C, whereas the proteasome subunit alpha type 2 was induced in both high temperature treatments. All genes belonging to the process of ubiquitin mediated proteolysis, except for the ubiquitin-like modifier-activating enzyme 1, were exclusively up-regulated at high PAR 17°C. The ubiquitin-like modifier-activating enzyme 1 was up regulated at both high temperature treatments, but featuring an enhanced up-regulation within the high light condition. Transcripts annotated as an ATP-dependent protease ATPase subunit and a cathepsin b-like cysteine proteinase 5 were induced at high PAR 17°C, whereas transcripts for the ATP-dependent clp protease proteolytic subunit was found to be up-regulated at high PAR 2°C. Interestingly we also detected two transcripts belonging to the family of cysteine proteases; the transcript abundance of a cysteine proteinase 2 was significantly enhanced at high PAR 17°C, whereas the transcript of a papain family cysteine protease containing protein was 2.3- fold induced at low light 17°C and 9.2-fold induced at high light 17°C.

## Discussion

This study represents the first broad scale gene expression study of a kelp species in response to abiotic stress. Genes covering about 70% of the genome were analyzed. Our aim was to investigate expression profile changes in *Saccharina latissima* after exposure to high PAR and temperature stress. We observed a multitude of transcriptional changes: Up to 30% of genes showed an altered expression after the exposure experiments. Similarly, in *Chondrus crispus* 25% of the studied genes exhibited transcriptional changes after exposure to different abiotic stressors [50]. In *Ectocarpus siliculosus,* almost 70% of the expressed genes featured significant changes in transcript abundance in response to stress [51]. We found that more genes were differently up-regulated than down-regulated, additionally more genes were regulated at high than at low PAR conditions; the largest amount of transcriptional changes was observed at high PAR and 17°C.

### Photosynthesis

Sporophytes exposed to high photosynthetically active radiation conditions showed a significant decrease in the maximum quantum yield of PS II. The combination of high PAR and a temperature of 17°C resulted in the highest degree of photoinhibition, showing 90% reduced *F*v/*F*m as compared to the control after 24 h of exposure. This was reflected by strong down-regulation (up to 60-fold) of transcripts responsible for photosynthetic components, e.g. encoding light harvesting complex proteins, photosystem II related proteins, porphyrin and chlorophyll metabolism proteins, and carbon fixation enzymes. Fluorescence measurements as well as gene expression studies indicate a breakdown of photosynthesis. Thus we suggest that *S. latissima* from the Arctic is particular susceptible to chronic photoinhibition at high PAR high temperature conditions. Preliminary experiments demonstrated that thalli exposed to high PAR and a temperature of 17°C for extended periods did not recover during a 6 h dark period of a 18 h light: 6 h dark cycle. This treatment seems to lead to irreversible damage. However, to confirm the hypothesis of chronic photoinhibition of an Arctic ecotype of *S. latissima* additional studies with temperate populations are required.

High PAR at 2°C caused a decrease in maximum quantum yield between 40–50% compared to the control, on the transcriptional level we observed an up-regulation between 2–5 fold of photosystem II, thylakoid, and light harvesting complex protein correlated transcripts. The low PAR treatments on the contrary induced no significant changes in maximum quantum yield of photosystem II, nevertheless light harvesting complex transcripts as well as some of the photosystem II transcripts were up-regulated at 2°C and down-regulated at 17°C with a fold change of about 2. Our results are in agreement with physiological studies showing that *Saccharina latissima* is well adapted to low temperature and low light conditions [13], [18], [52]–[54]. The observed effect of high PAR stress on the photosynthesis of *S. latissima* might be due to the shade adaption of the species, which is expressed in a low compensation point, high absorbance and photosynthetic efficiency (α) especially in shade grown plants [55], [56], a physiological constitution favoring strong photoinhibition when exposed to high PAR [57]. Low light adapted subtidal algae are more sensitive to high PAR stress than high irradiance adapted algae [58]. Another study revealed that high temperatures can enhance photodamaging effects of high PAR in plants [59]. In *Saccharina latissim*a photosynthetic efficiency decreases with increasing temperatures [7]. However, hyper-optimal temperatures of up to 22°C did not influence photoinhibition in *S. latissima* [60]. These contradicting results might be due to ecotypic differentiation within the species, in turn explaining the huge capacity for climatic adaption and therefore its wide geographic distribution.

The observed regulation of transcripts encoding the light harvesting complex (LHC) in all our experimental conditions leads to the suggestion, that the regulation of LHC proteins is a prominent part of the photoacclimatory mechanism in *S. latissima*. This agrees with former studies, showing that changes in light availability and temperature, as well as the age of the thalli, influenced the pigment content and composition in *S. latissima* [7], [21], [22]. The expression of the nuclear encoded LHC genes is regulated by chloroplast redox signals, including the redox state of electron transport components [34] as well as chlorophyll biosynthetic intermediates [61]. The abundance of transcripts correlated to photosystem II varied within the stress treatments, with a trend towards up-regulation at low temperatures and down-regulation at high temperatures. The photosystem II biogenesis protein psp29 was up-regulated at both low temperature conditions with higher transcript abundance at high PAR. Disruption of this gene results in an impairment of PS II function under high irradiance confirming the involvement of psp29 in PS II biogenesis and disintegration following photodamage [62]. Hence the up-regulation of this gene at low temperatures might be due to a cold shock photoinhibition effect, as temperature leads to an increase of photo-oxidative stress, partly because of the reduced activity of the Calvin cycle [34], [63].

Furthermore we detected specific transcriptional changes for those treatments causing decreased maximum quantum yield: photosystem II D2 protein was up-regulated at high PAR 2°C, whereas photosystem II reaction center protein D1/psbA was strongly down-regulated at high PAR 17°C. Photoinhibition is a state of physiological stress that appears when excessive light is absorbed by the photosynthetic apparatus; chronic photoinhibition occurs when the rate of damage exceeds the capacity of the PSII repair and is associated with the inactivation and degradation of PS II reactions centers [21], [64]–[66]. The recovery mechanisms of photosystem II include the removal of damaged D1 proteins and replacement by newly synthesized molecules for the PSII [67]. Recent studies by [68] revealed that photodamage of PS II is not only associated with damage within the PSII reaction center, but also with a depressed repair rate caused by inhibition of the synthesis of the D1 protein at the step of protein translation. Our finding of strongly lowered maximum quantum yield and reduced transcript abundance of D1 therefore suggest that the severe damage to PS caused by the 17°/high PAR treatment could not be overcome by transcriptional regulation in *S. latissima*.

### General stress response characteristics

*S. latissima* exhibited significant changes in gene expression after the exposure to different temperatures, even though no influence on the maximum quantum yield of photosynthesis was observed. The higher number of high temperature-responsive genes indicates that high temperature had stronger effects on the gene expression in *S. latissima* than low temperature. This implies that high temperatures are more harmful for *S. latissima* than low temperatures, resulting in stronger efforts to overcome the negative effects. Gene enrichment analysis showed down-regulation of carbohydrate biosynthetic and catabolic processes after exposure to high temperature, as well as repression of transcripts correlated to photosynthesis and carbon fixation. A possible explanation might be the increasing reaction rate of enzymes at elevated temperatures. In many cellular processes the reaction rate tends to increase with a Q_10_ of about 2 [69]. Furthermore, a down-regulation of transcripts can partially be caused by energetic and mass limitations of the full transcriptome, favoring transcripts of acute and chronic stress response.

At 2°C we detected up-regulation of genes correlated with the metabolism of glycine, serine, and threonine. An increase in the amino acid content during cold acclimation was also demonstrated for the green algae *Klebsormidium flaccidum* [70]. Only few studies were conducted on the amino acid metabolism of macroalgae [71], [72] and until now no data are available on the influence of cold acclimation on the amino acid metabolism of macroalgae. Serine is involved in the final step of cysteine biosynthesis, which includes incorporating of a sulfide moiety and an amino acid moiety from serine through O-acetyl-L serine [73]. The amino acids cysteine, glycine, and glutamate are essential for synthesis of Glutathione (GSH), a reducing co-factor for several enzymes involved in ROS detoxification [49], [74]. Furthermore it was observed that GSH levels respond to the availability of cysteine and glycine, an elevated capacity for GSH synthesis in the chloroplast can influence metabolic upstream events for satisfying the increased substrate demand [75]. The up-regulation of glycine, serine, and threonine metabolism in *S. latissima* at low temperature thus might be reflecting the higher demand of GSH due to an increase of photooxidative stress. However, although mRNA levels often indicate active metabolic patterns, in some cases it is not possible to extrapolate the enzymatic activity from the transcriptional level. Future investigations should therefore combine transcriptional studies with measurements of particular down-stream components.

The interactive effects of temperature and light stress on photosynthesis in *S. latissima* were also observed at the transcriptional level; the amount of high PAR responsive ESTs doubled at high temperature. In general high PAR caused an up-regulation of catabolic processes for energy supply as well as for genes with antioxidant functions. The combination of light and low temperature stress led on the one hand to an induction of lipid catabolism and carbohydrate metabolism, on the other hand to an increased number of induced gene activities in cellular amino acid biosynthesis, cellular nitrogen compound biosynthesis and nucleobase biosynthesis. Furthermore we observed enhanced up-regulation of the aminoacyl-tRNA metabolism. These results suggest that energy was generated for removal and replacement of damaged proteins as well as for the synthesis of stress related proteins, e.g. Hsps and ROS scavenging enzymes. Cells are able to balance finely the expression of stress related and growth related genes. Stress results in an induction of genes with energy generating, heat shock and antioxidant functions, whereas growth related genes are mostly repressed [76]. Few studies focused on molecular stress response patterns in other macroalgae. [77] Collén et al. 2006 examined the gene expression profiles of *Chondrus crispus* after exposure to methyl jasmonate that resulted in a repression of genes involved in energy conversion and general metabolism and to an induction of stress-related genes, e.g. glutathione S-transferase and Hsp-20. In another study Collén et al. 2007 investigated the response of the transcriptome of *Chondrus crispus* to high light, high temperature and osmotic stress. At these conditions decreased expression of energy- and protein synthesis and increased expression of stress genes was observed, suggesting that available resources were rather used for reducing potential damage and repair of structure than for growth related processes [50]. A recent study of global gene expression in *Ectocarpus siliculosus* in response to abiotic stress gave similar results, namely repression of genes related to growth and primary metabolism and induction of energy generation and production of protectants [51].

Excessive light at high temperature was the most destructive stress condition for *S. latissima*, resulting in a strong down-regulation of several metabolic processes, e.g. photosynthesis, carbohydrate metabolism, and amino acid metabolism. We detected enhanced expression of ROS scavengers as well as a high abundance of Hsp transcripts. A striking feature of the high PAR/high temperature treatment was the significant induction of several genes involved in proteolysis, e.g. protease regulatory subunits and components of the ubiquitin-mediated proteolysis, which did not occur in any other of our stress treatments. Our data suggest that this treatment caused the highest degree of protein dysfunction. The 26S proteasome is an essential part of the ATP-dependent proteolysis in eukaryotes, consisting of two functionally different sub-complexes, the 20S core protease and a 19S regulatory particle [78], [79]. It is responsible for the degradation of various cellular proteins, including also critical regulatory proteins (e.g. cyclins) as well as transcription factors; hence it is involved in various cellular processes, for instance apoptosis, metabolic regulation and signal transduction [80], [81]. Interestingly the induction of two genes annotated as different cysteine proteases, cysteine proteinase 2 and papain family cysteine protease containing protein was observed in *S. latissima* under excessive light and high temperature. Cysteine proteases are involved in intracellular protein degradation, they respond to different internal and external stimuli and are able to provide up to 90% of the proteolytic activity [82], [83]. Moreover, several studies showed that cysteine protease activity is a key event in programmed cell death or apoptosis in plants and different phytoplankton species [84]–[87]. Taken all together, our data indicate that *S. latissima* is not able to adapt to excessive light in connection with high temperatures. Further studies are needed to distinguish between apoptosis versus necrosis as the major process in *S. latissima* at high temperatures and under high light.

### ROS scavenging enzymes

We detected significant induction of several transcripts correlated with reactive oxygen species (ROS) scavenging mechanisms. Transcripts coding for superoxide dismutase (SOD) [Fe] were induced at low PAR 17°C, whereas transcripts encoding the mitochondrial SOD precursor were up-regulated in both light conditions at 17°C. ROS species such as superoxide and hydrogen peroxide are involved in response to biotic and abiotic stress in macroalgae [39], [88]–[90]. SOD catalyzes the reaction from superoxide anion to hydrogen peroxide and oxygen [29], [91], [92]. A couple of studies focused on the SOD enzyme activity in response to abiotic stresses in macroalgae. Bischof et al. (2003) detected an increased enzyme activity of SOD in *Ulva* after exposure to PAR and UV irradiance [29], the same was shown for *Chaetomorpha linum* after exposure to UV radiation [93]. An increased SOD activity was also observed in *Ulva fasciata* in response to salinity stress [94]. Gene expression analysis of *Chondrus crispus* indicated an up-regulation of SOD transcripts after exposure to methyl jasmonate [77]. Since the generation of ROS increases at low temperature [43] the observed up-regulation of SOD at high temperature in our experiments seems to be counterintuitive at first sight. However, our results agree with other investigations: Vyas & Kumar (2005) analyzed the activity of SOD from tea (*Camellia sinensis*) over a wide temperature range and detected increased activity of SOD at decreasing temperatures with an optimum at 0°C. This peculiarity was also shown for several phytoplankton species by Perelman et al. (2006), which suggested that the benefit of this low temperature activation provides a faster protection than de novo enzyme synthesis alone [32].

We observed significant induction of genes coding for enzymes of the ascorbate-glutathione cycle. Ascorbate peroxidase (APX) was induced after exposure to high PAR at 2°C, whereas the glutathione reductase (GR) was up-regulated in all treatments except low PAR at 17°C, with the strongest induction after exposure to high PAR at 2°C. Additionally, a higher number of transcripts coding for glutaredoxin was found in response to high temperature. The ascorbate-glutathione cycle is considered to play an important role in ROS scavenging [46]. In this pathway reduced glutathione (GSH) is required for the reduction of dehydroascorbate, which is generated via monodehydroascorbate by APX activity [95]. Glutaredoxins are small heat-stable disulphide oxidoreductases, which catalyze glutathione–dependent reactions and are suggested to protect cells against oxidative damage [96], [97]. However, plants contain various glutaredoxins whose functions are still unknown [98]. Some glutaredoxins were shown to be up-regulated by heat stress [99], furthermore there is evidence that glutaredoxins interact with Hsps and antioxidant enzymes [100], [101].

In macroalgae the activity of ROS scavenging enzymes has been studied extensively. Macroalgae exhibit increased activity of GR and APX after exposure to various abiotic factors such as low temperature [102], UV-radiation [48], [90], copper concentration [39], [47] and desiccation [95]. Only a few projects focused on gene expression in macroalgae during stress. Collén *et* *al.* 2007 observed the over expression of APX transcripts in *Chrondrus crispus* after exposure to high light conditions [50]. The green alga *Ulva fasciata* showed enhanced up-regulation of APX and GR genes in response to copper stress [47]. Dittami *et* *al.* (2009) detected enhanced expression of glutaredoxin and glutathione peroxidase in *Ectocarpus siliculosus* after exposure to oxidative and hyper saline stress, respectively [51]. Our results conform to previous studies, demonstrating that the ascorbate-glutathione cycle is an important anti-oxidant mechanism in macroalgae.

Two genes of the chloroplastic thioredoxin system were detected among the significantly up-regulated ones. Transcripts for chloroplastic thioredoxin showed enhanced abundance at high PAR conditions, whereas thioredoxin reductase was induced after all treatments, except for low PAR at 17°C, with highest fold change detected after exposure to high PAR at 2°C. Thioredoxins (Trx) are low molecular weight thiol-disulphide oxidoreductases and involved in redox homeostasis [103], [104]. Thioredoxin reductase is an abundant thiol based peroxidase, which catalyzes the reduction of thioredoxin [105]. Two isoforms of thioredoxin reductase are present in the chloroplast, which use either ferredoxin or NADPH as an electron donor [103]. Plastidic thioredoxins are induced in response to high light intensities, indicating a function in redox balancing related to photosynthesis [106]. They regulate photosynthetic enzymes by light via ferredoxin-thioredxin reductase, and are critical for redox regulation of protein function and signaling via thio redox control [107]. Furthermore studies by Michelet et al. [108] suggest a complex interaction between Trx, glutaredoxins and glutathione, which are key components of the cellular redox-signaling network. Taken together it can be concluded that *S. latissima* possesses strong protection mechanisms against oxidative damage. ROS scavenging mechanisms showed the strongest induction after exposure to high light at 2°C, suggesting that the highest amount of ROS is generated under excessive light in combination with low temperature.

### Heat shock proteins

We observed differential up-regulation of various heat shock proteins (Hsps). Members of the Hsp 70 family were most strongly expressed after the high PAR 17°C treatment. Interestingly three Hsps, Hsp 33, Hsp 60, and Hsp 90, respectively, were exclusively induced at low temperatures. Another four transcripts (Heat shock 70 kda protein 4, Heat shock cognate 70 kda protein 1, Heat shock factor-binding protein 1, mitochondrial heat shock protein mthsp70) were solely up-regulated in response to high temperature in combination with high PAR. Hsps are highly conserved proteins, which are not only important for a broad array of normal cellular processes, but more so play a crucial role in response to stress by re-establishing functional protein conformation and thus cellular homeostasis [109]–[111]. We detected members of four families, small heat shock proteins (sHsps), Hsp 60, Hsp 70, and Hsp90, among up-regulated genes. Until now, little is known about the function of Hsps in macroalgae in response to stress. Most studies focused on the expression of Hsp 70, which was shown to be heat shock induced in the red alga *Plocamium cartilagineum* [112], the brown alga *Laminaria japonica* [111] and the green alga *Ulva pertusa* [113]. Collén *et* *al.* 2007 detected in the red algae *Chrondrus crispus* an enhanced transcript abundance of Hsp 70 in response to high light and high temperature, furthermore a high light dependent induction of the Hsp 90 was observed [50]. One study investigated the survival under heat stress in intertidal embryos of *Fucus spp.*; here the Hsp 60 showed a higher expression level at elevated temperatures [114]. It was recently shown by Pearson et al. (2010) that temperature stress led to an enhanced expression of several different Hsps in *Fucus*, including the Hsp 18, Hsp 70, Hsp 83 as well as the Hsp STI [115]. Our data suggests that the sophisticated regulation of Hsps in *S. latissima* is a prominent part of acclimation not only to temperature but also to combined environmental stresses like high PAR in combination with high temperature.

### Conclusions

Our study shows that *S. latissim*a from the Arctic (Spitsbergen) responds to high temperature and light stress with a multitude of transcriptional changes, and that high temperature (17°C) had stronger effects on the gene expression in *S. latissima* than low temperature (2°C). We conclude that *S. latissima,* as a cold adapted species, requires a larger degree of metabolic reorganization for acclimating to high temperatures than to low temperatures. For all parameters measured, the combination of high temperatures with high light intensities caused the strongest response and proved most harmful for the alga; even leading to an up-regulation of programmed cell death related genes. The combination of the stress factors light and temperature led to interactive effects on photosynthesis and gene expression profiles. Thus simultaneous influence of several stress factors can elevate their damaging effects, and might lead to an increase of susceptibility to additional stresses [116], [117]. Possible consequences of this reduced resilience for kelps include local extinctions of population near the southern distribution limit as well as a shift in its distribution towards the Arctic [118]. Future studies should therefore combine more and additional factors, e.g. enhanced CO_2_ concentrations, changing salinity, or enhanced UV-radiation. With respect to the ecotypic differentiation within this species, similar studies on gene expression under abiotic stresses should be conducted with *S. latissima* populations of the southern distribution boundary, to gain further insights into variability and acclimation potential among spatially separated populations.

## Materials and Methods

### Culture conditions and stress treatments

*Saccharina latissima* (Lane) sporophytes were raised from gametophyte cultures, which were established from spores of fertile sporophytes collected by SCUBA diving in Kongsfjorden, (79°N; 11°E; Svalbard, Norway; AWI culture numbers: 3123, 3124). Male and female gametophytes were fragmented together, transferred to petri dishes filled with Provasoli enriched Seawater (PES) [119] and cultured at 10±1°C and 30 µmol photons m^2^ s^−1^ photosynthetically active radiation (PAR) at 18 h light∶6 h dark period. After 2 weeks developing sporophytes were transferred to aerated 5l culture bottles and grown in PES until they reached a size of 5–7 cm. The medium was changed twice per week.

Young sporophytes were exposed for 24 h in environmentally controlled rooms set to 2, 12, and 17°C±1°C to high photosynthetically active radiation (107.8±5 µmol photons m^2^ s^−1^) and low photosynthetically active radiation (23.8±3.1 µmol photons m^2^ s^−1-^). For determining the light intensities of the stress treatments preliminary experiments with different irradiances were conducted. All experiments were conducted with five biological replicates. Photosynthetically active radiation (PAR) was provided by Osram daylight fluorescent tubes (Biolux, 36W; Osram, Germany), and was determined by using a LI- 250 light meter (LI-COR, Lincoln; USA).

### Fluorescence measurements

Photosynthetic efficiency was measured to determine the extent of photoinhibition in response to photosynthetically active radiation (PAR) and temperature stress. The maximum quantum yield of PS II (*F*v/*F*m) was determined in the beginning and at the end of the experiment by use of an Imaging PAM (Pulse Amplitude Fluorometer, Walz, Effeltrich, Germany). Sporophytes were dark-adapted for 5 min prior to the measurements. After the fluorescence measurements the sporophytes were frozen in liquid nitrogen and stored at −80°C until further use.

*F*v/*F*m values of sporophytes obtained under the different conditions were analyzed using a two-way ANOVA with repeated measurements (p<0.01). Significant differences and interaction of means were compared with the post hoc Tukey test (HSD, p<0.01). All statistical analyses were done using SPSS software version 19 (IBM, USA).

### RNA Extraction

Frozen sporophytes were ground in liquid nitrogen and transferred to 2.0 ml Eppendorf tubes. 1 ml extraction buffer (2% CTAB, 1 M NaCl, 100 mM Tris pH 8, 50 mM EDTA, pH 8) and 20 μl DTT 2M were added and mixed well. The mixture was incubated at 45°C for 10 min. One volume of chloroform: isoamylalcohol (24∶1) was added and mixed vigorously for 10 min. The tubes were centrifuged for 20 min at 20°C and 12 000 g. 750 μl of the aqueous phase were transferred into a new tube. 0.3 volumes of EtOH 100% were added and mixed gently by inverting the tube. One volume of chloroform: isoamylalcohol (24∶1) was added and a second chloroform extraction followed. 500 μl of the supernatant were transferred to a new cup and total RNA was extracted using a Quiagen Plant Mini Kit (Qiagen, Hildesheim; Germany) according to manufactures protocol for RNA Extraction including on-column DNA-digestion. Quantity and purity of the extracted RNA was determined by a NanoDrop ND-1000 spectrometer (PeqLab, Erlangen, Germany). integrity of the RNA was verified by Nano Chip Assay with the 2100 Bioanalyzer device (Agilent Technologies, Böblingen, Germany).

### Microarray design and hybridization

2×105 k microarrays slides were designed with Agilent's eArray online application tool containing 60mer oligonucleotides probes, which were designed from a *Saccharina latissima* cDNA library, thereby 26,224 transcripts were represented by 3 individual probes. The cDNA library, featuring functional genome coverage of approximately 70%, was established from RNA sampled under various light and temperature regimes [120]. Original sequencing files of the cDNA library were uploaded at the NCBI Sequence Read Archive (http://www.ncbi.nlm.nih.gov/sra; accession number SRR305166). Transcripts of the cDNA library were annotated with Blast2GO [121] and blasted against NCBI non-redundant protein database (http://blast.ncbi.nlm.nih.gov/Blast.cgi, NCBI-nr) and the Swiss-Prot protein knowledgebase (http://www.uniprot.org/) (release 2010_7) using the BLASTX algorithm with an e-value cut-off of 10^−7^. KOG categories for the cDNA library were determined within the KOG/euNOG sets of orthologies taken from eggNOG (Version 3.0 [122]) by trpsblastn [123] with an e-value cut-off of 10^−10^.

Total RNA of stress treatments (low PAR 2° and 17°C, high PAR 2° and 17°C) was hybridized against the control treatment (low PAR 12°C), consisting of pooled RNA of 4 individuals. Hybridizations were carried out in 4 biological replicates, each hybridized on a single microarray. For total RNA labeling the Agilent two-color low RNA Input Linear Amplification kit (Agilent Technologies, Waldbronn, Germany) was used. RNA from control treatments was labeled by fluorescent complementary RNA (cRNA) synthesis with cyanine-3-CTP, stress treatment RNA was labeled with cyanine5-CTP.

Prior to the labeling, the Agilent RNA Spike-In Mix (Agilent) was added to 700 ng of total RNA. Due the extensive length of 3′ untranslated regions (UTRs) occurring in brown algae cDNA synthesis was performed using a blend of T7 promoter primer and T7 nonamer primer used in equal molarity. cRNA synthesis and purification of labeled RNA was performed following the Agilent Low RNA Input Linear Amplification Kit protocol (Agilent). cRNA yield and specific activity of cyanine-3 and cyanine-5 was determined using the NanoDrop ND-1000 spectrometer (PeqLab, Erlangen, Germany).

Hybridizations were performed with 825 ng of cyanine-3 and cyanine-5 labeled cRNA for 17 h at 65°C. Afterwards microarray disassembly and wash procedure followed according to manufacturer's instructions (Agilent). Microarrays were scanned with the Agilent G2565AA scanner. Raw data processing was carried out with the Agilent Feature Extraction Software version 9.1.3.1 (FE), for quality monitoring of the microarrays the Agilent QC Tool (v1.0) with the metric set GE2_QCMT_Feb07 was used. Normalization was conducted with the Agilent Feature Extraction Software version 9.1.3.1 (FE), which employs a linear normalization correction and the Lowess algorithm.

The microarray design, raw data and normalized data as well as the detailed experimental design are MIAME compliant and deposited in a MIAME compliant database (ArrayExpress at the EBI; http://www.ebi.ac.uk/microarray-as/ae/; ID: E-MEXP-3450).

### Statistical analysis of microarray data

Differential gene expression was analyzed using the GeneSpring GX software platform version 11 (Agilent) with the implemented statistical tests. An ANOVA was performed, followed by a post hoc test Tukey HSD with the Benjamini Hochberg FDR correction. Genes were considered to be differentially expressed when p-Values were less than 0.01 and the calculated fold changes between the control and the treatment exceeded a value of 2.

To perform statistical assessments of GO annotations, whose abundance is significantly different between the regulated genes within the various exposure treatments and the whole microarray, gene set enrichment analysis were done using Blast2GO [121]. Blast2GO employs the Fisher's exact test including corrections for multiple testing using FDR (false discovery rate), FWER (family-wise error rate) and single test *p*-value (p<0.01). Additionally significantly enriched KEGG pathways were identified with KOBAS (http://kobas.cbi.pku.edu.cn/home.do) using a hypergeometric test (p<0.01).

## Supporting Information

Table S1 **Full list of over-represented Gene Ontology terms for all sections of the Venn diagrams.**
(XLS)

Table S2 **Full list of over-represented Gene Ontology terms for all stress conditions.**
(XLS)

Table S3 **Full list of genes contributing to the results of the KOBAS analyses.**
(XLS)

Table S4 **Full list of regulated ESTs with annotations for all stress conditions.**
(XLS)

## References

[1] Lüning K (1990) Seaweeds. Their environment, biogeography and ecophysiology. New York: Wiley & Sons Inc.

[2] Roleda M, Wiencke C, Hanelt D, Bischof K (2007) Sensitivity of the early life stages of macroalgae from the northern hemisphere to ultraviolet radiation. Photochemistry and Photobiology 83: 851–862. 17645656 10.1562/2006-08-17-IR-1005

[3] Dayton PK (1985) Ecology of kelp communities. Annual Review of Ecology and Systematics 16: 215–245.

[4] Smith SV (1981) Marine macrophytes as a global carbon sink. Science 211: 838–840. 17740399 10.1126/science.211.4484.838

[5] Charpy-Roubaud C, Sournia A (1990) The comparative estimation of phytoplanktonic, microphytobenthic and macrophytobenthic primary productions in the oceans. Marine Microbial Food Webs 4: 31–57.

[6] Carlsen B, Johnsen G, Berge J, Kuklinski P (2007) Biodiversity patterns of macro-epifauna on different lamina parts of *Laminaria digitata* and *Saccharina latissima* collected during spring and summer 2004 in Kongsfjorden, Svalbard. Polar Biology 30: 939–943.

[7] Davison I, Greene M, Podolak E (1991) Temperature acclimation of respiration and photosynthesis in the brown alga *Laminaria saccharina* . Marine Biology 110: 449–454.

[8] Kirst G, Wiencke C (1995) Ecophysiology of polar algae. Journal of Phycology 31: 181–199.

[9] Ensminger I, Busch F, Huner NPA (2006) Photostasis and cold acclimation: sensing low temperature through photosynthesis. Physiologia Plantarum 126: 28–44.

[10] Gómez I, Wulff A, Roleda MY, Huovinen P, Karsten U, et al (2009) Light and temperature demands of marine benthic microalgae and seaweeds in polar regions. Botanica Marina 52: 593–608.

[11] Wiencke C, Roleda MY, Gruber A, Clayton MN, Bischof K (2006) Susceptibility of zoospores to UV radiation determines upper depth distribution limit of Arctic kelps: evidence through field experiments. Journal of Ecology 94: 455–463.

[12] Bartsch I, Wiencke C, Bischof K, Buchholz C, Buck B, et al (2008) The genus *Laminaria sensu lato*: recent insights and developments. European Journal of Phycology 43: 1–86.

[13] Hanelt D (1998) Capability of dynamic photoinhibition in Arctic macroalgae is related to their depth distribution. Marine Biology 131: 361–369.

[14] Graham MH, Kinlan BP, Druehl LD, Garske LE, Banks S (2007) Deep-water kelp refugia as potential hotspots of tropical marine diversity and productivity. Proceedings of the National Academy of Sciences 104: 16576–16580. 10.1073/pnas.0704778104 PMC2034254 17913882

[15] Bolton JJ, Germann I, Lüning K (1983) Hybridization between Atlantic and Pacific representatives of the simplices section of *Laminaria* (Phaeophyta). Phycologia 22: 133–140.

[16] Borum J, Pedersen M, Krause Jensen D, Christensen P, Nielsen K (2002) Biomass, photosynthesis and growth of *Laminaria saccharina* in a high-arctic fjord, NE Greenland. Marine Biology 141: 11–19.

[17] Gerard VA (1988) Ecotypic differentiation in light-related traits of the kelp *Laminaria saccharina* . Marine Biology 97: 25–36.

[18] Gerard VA, Dubois KR (1988) Temperature ecotypes near the southern boundary of the kelp *Laminaria saccharina* . Marine Biology 97: 575–580.

[19] Gerard VA (1990) Ecotypic differentiation in the kelp *Laminaria saccharina*: phase-specific adaptation in a complex life-cycle. Marine Biology 107: 519–528.

[20] Müller R, Wiencke C, Bischof K (2008) Interactive effects of UV radiation and temperature on microstages of Laminariales (Phaeophyceae) from the Arctic and North Sea. Climate Research 37: 203–213.

[21] Hanelt D, Wiencke C, Karsten U, Nultsch W (1997) Photoinhibition and recovery after high light stress in different developmental and life-history stages of *Laminaria saccharina* (Phaeophyta). Journal of Phycology 33: 387–395.

[22] Machalek K, Davison I, Falkowski P (1996) Thermal acclimation and photoacclimation of photosynthesis in the brown alga *Laminaria saccharina* . Plant, Cell & Environment 19: 1005–1016.

[23] Gévaert F, Créach A, Davoult D, Migne A, Levavasseur G, et al (2003) *Laminaria saccharina* photosynthesis measured in situ: photoinhibition and xanthophyll cycle during a tidal cycle. Marine Ecology-Progress Series 247: 43–50.

[24] Han TJ, Kain JM (1996) Effect of photon irradiance and photoperiod on young sporophytes of four species of the Laminariales. European Journal of Phycology 31: 233–240.

[25] Crépineau F, Roscoe T, Kaas R, Kloareg B, Boyen C (2000) Characterisation of complementary DNAs from the expressed sequence tag analysis of life cycle stages of *Laminaria digitata* (Phaeophyceae). Plant Molecular Biology 43: 503–513. 11052202 10.1023/a:1006489920808

[26] Roeder V, Collén J, Rousvoal S, Corre E, Leblanc C, et al (2005) Identification of stress gene transcripts in *Laminaria digitata* (Phaeophyceae) protoplast cultures by expressed sequence tag analysis. Journal of Phycology 41: 1227–1235.

[27] Cosse A, Potin P, Leblanc C (2009) Patterns of gene expression induced by oligoguluronates reveal conserved and environment-specific molecular defense responses in the brown alga *Laminaria digitata* . New Phytologist 182: 239–250. 19192194 10.1111/j.1469-8137.2008.02745.x

[28] Qureshi M, Qadir S, Zolla L (2007) Proteomics-based dissection of stress-responsive pathways in plants. Journal of Plant Physiology 164: 1239–1260. 17662502 10.1016/j.jplph.2007.01.013

[29] Bischof K, Janknegt P, Buma A, Rijstenbil J, Peralta G, et al (2003) Oxidative stress and enzymatic scavenging of superoxide radicals induced by solar UV-B radiation in *Ulva* canopies from southern Spain. Scientia Marina 67: 353–359.

[30] Janknegt P, van de Poll W, Visser R, Rijstenbil J, Buma A (2008) Oxidative stress responses in the marine Antarctic diatom *Chaetoceros brevis* (Bacillariophyceae) during photoacclimation. Journal of Phycology 44: 957–966. 27041614 10.1111/j.1529-8817.2008.00553.x

[31] Bischof K, Rautenberger R (2012) Seaweed responses to environmental stress: Reactive oxygen and antioxidative strategies. In: Wiencke CB, K., editor. Recent advances in Seaweed Biology. Berlin, Heidelberg: Springer Verlag.

[32] Perelman A, Dubinsky Z, Martinez R (2006) Temperature dependence of superoxide dismutase activity in plankton. Journal of Experimental Marine Biology and Ecology 334: 229–235.

[33] Kaplan F, Kopka J, Sung DY, Zhao W, Popp M, et al (2007) Transcript and metabolite profiling during cold acclimation of *Arabidopsis* reveals an intricate relationship of cold-regulated gene expression with modifications in metabolite content. Plant Journal 50: 967–981. 17461790 10.1111/j.1365-313X.2007.03100.x

[34] Pfannschmidt T (2003) Chloroplast redox signals: how photosynthesis controls its own genes. Trends in Plant Science 8: 33–41. 12523998 10.1016/s1360-1385(02)00005-5

[35] Timperio A, Egidi M, Zolla L (2008) Proteomics applied on plant abiotic stresses: role of heat shock proteins (HSP). Journal of Proteomics 71: 391–411. 18718564 10.1016/j.jprot.2008.07.005

[36] Panchuk II, Volkov RA, Schoffl F (2002) Heat stress- and heat shock transcription factor-dependent expression and activity of ascorbate peroxidase in *Arabidopsis* . Plant Physiology 129: 838–853. 12068123 10.1104/pp.001362 PMC161705

[37] Dring MJ (2006) Stress resistance and disease resistance in seaweeds: The role of reactive oxygen metabolism. In: Callow JA, editor. Advances in Botanical Research, Vol 43: Incorporating Advances in Plant Pathology. London: Academic Press Ltd-Elsevier Science Ltd. 175–207.

[38] Lesser MP (2006) Oxidative stress in marine environments: biochemistry and physiological ecology. Annual Review of Physiology 68: 253–278. 10.1146/annurev.physiol.68.040104.110001 16460273

[39] Contreras L, Mella D, Moenne A, Correa JA (2009) Differential responses to copper-induced oxidative stress in the marine macroalgae *Lessonia nigrescens* and *Scytosiphon lomentaria* (Phaeophyceae). Aquatic Toxicology 94: 94–102. 19581008 10.1016/j.aquatox.2009.06.004

[40] Kumar M, Kumari P, Gupta V, Reddy CRK, Jha B (2010) Biochemical responses of red alga *Gracilaria corticata* (Gracilariales, Rhodophyta) to salinity induced oxidative stress. Journal of Experimental Marine Biology and Ecology 391: 27–34.

[41] Lehmann M, Schwarzlander M, Obata T, Sirikantaramas S, Burow M, et al (2009) The metabolic response of *Arabidopsis* roots to oxidative stress is distinct from that of heterotrophic cells in culture and highlights a complex relationship between the levels of transcripts, metabolites, and flux. Molecular Plant 2: 390–406. 19825624 10.1093/mp/ssn080

[42] Foyer CH, Noctor G (2005) Oxidant and antioxidant signalling in plants: a re-evaluation of the concept of oxidative stress in a physiological context. Plant Cell and Environment 28: 1056–1071.

[43] Asada K (1997) The role of ascorbate peroxidase and monodehydroascorbate reductase in H2O2 scavenging in plants. In: JG S, editor. Oxidative stress and the molecular biology of antioxidant defenses. New York: Cold Spring Harbor Laboratory Press.

[44] Asada K (1999) The water-water cycle in chloroplasts: Scavenging of active oxygens and dissipation of excess photons. Annual Review of Plant Physiology and Plant Molecular Biology 50: 601–639. 10.1146/annurev.arplant.50.1.601 15012221

[45] Apel K, Hirt H (2004) Reactive oxygen species: Metabolism, oxidative stress, and signal transduction. Annual Review of Plant Biology 55: 373–399. 10.1146/annurev.arplant.55.031903.141701 15377225

[46] Noctor G, Foyer CH (1998) Ascorbate and glutathione: keeping active oxygen under control. Annual Review of Plant Physiology and Plant Molecular Biology 49: 249–279. 10.1146/annurev.arplant.49.1.249 15012235

[47] Wu T-M, Lee T-M (2008) Regulation of activity and gene expression of antioxidant enzymes in *Ulva fasciata* Delile (Ulvales, Chlorophyta) in response to excess copper. Phycologia 47: 346–360.

[48] Shiu C-T, Lee T-M (2005) Ultraviolet-B-induced oxidative stress and responses of the ascorbate-glutathione cycle in a marine macroalga *Ulva fasciata* . Journal of Experimental Botany 56: 2851–2865. 16157654 10.1093/jxb/eri277

[49] Noctor G (2006) Metabolic signalling in defence and stress: the central roles of soluble redox couples. Plant Cell and Environment 29: 409–425. 10.1111/j.1365-3040.2005.01476.x 17080595

[50] Collén J, Guisle-Marsollier I, Leger J, Boyen C (2007) Response of the transcriptome of the intertidal red seaweed *Chondrus crispus* to controlled and natural stresses. New Phytologist 176: 45–55. 17803640 10.1111/j.1469-8137.2007.02152.x

[51] Dittami S, Scornet D, Petit J, Segurens B, Da Silva C, et al (2009) Global expression analysis of the brown alga *Ectocarpus siliculosus* (Phaeophyceae) reveals large-scale reprogramming of the transcriptome in response to abiotic stress. Genome Biology 10: 51. 10.1186/gb-2009-10-6-r66 PMC2718500 19531237

[52] Bolton JJ, Lüning K (1982) Optimal-growth and maximal survival temperatures of atlantic *Laminaria* species (Phaeophyta) in culture. Marine Biology 66: 89–94.

[53] Dunton KH (1985) Growth of dark-exposed *Laminaria saccharina* (L.) Lamour. and *Laminaria solidungula* J. Ag. (Laminariales, Phaeophyta) in the Alaskan Beaufort Sea. Journal of Experimental Marine Biology and Ecology 94: 181–189.

[54] Davison IR, Davison JO (1987) The effect of growth temperature on enzyme activities in the brown alga *Laminaria saccharina* . British Phycological Journal 22: 77–87.

[55] Adams WW, Osmond CB, Sharkey TD (1987) Responses of 2 CAM species to different irradiances during growth and susceptibility to photoinhibition by high light. Plant Physiology 83: 213–218. 16665205 10.1104/pp.83.1.213 PMC1056327

[56] Seemann JR, Sharkey TD, Wang JL, Osmond CB (1987) Environmental effects on photosynthesis, nitrogen-use efficiency, and metabolite pools in leaves of sun and shade plants. Plant Physiology 84: 796–802. 16665524 10.1104/pp.84.3.796 PMC1056672

[57] Anderson JM, Osmond CB (1987) Shade-sun responses: compromises between acclimation and photoinhibition. In: Kyle DO, CB; Arntzen, CJ, editor. Topics in Photosynthesis. Amsterdam Elsevier. 1–38.

[58] Häder DP, Figueroa FL (1997) Photoecophysiology of marine macroalgae. Photochemistry and Photobiology 66: 1–14.

[59] Ludlow MM (1987) Light stress at high temperature. In: Kyle DJ, Osmond CB AC, editors. Topics in Photosynthesis. Amsterdam Elsevier. 89–109.

[60] Bruhn J, Gerard V (1996) Photoinhibition and recovery of the kelp *Laminaria saccharina* at optimal and superoptimal temperatures. Marine Biology 125: 639–648.

[61] Surpin M, Larkin RM, Chory J (2002) Signal transduction between the chloroplast and the nucleus. Plant Cell 14: S327–S338. 12045286 10.1105/tpc.010446 PMC151264

[62] Keren N, Ohkawa H, Welsh EA, Liberton M, Pakrasi HB (2005) Psb29, a conserved 22-kD protein, functions in the biogenesis of photosystem II complexes in *Synechocystis* and *Arabidopsis* . Plant Cell 17: 2768–2781. 16155179 10.1105/tpc.105.035048 PMC1242271

[63] Haghjou MM, Shariati M, Smirnoff N (2009) The effect of acute high light and low temperature stresses on the ascorbate-glutathione cycle and superoxide dismutase activity in two *Dunaliella salina* strains. Physiologia Plantarum 135: 272–280. 19236661 10.1111/j.1399-3054.2008.01193.x

[64] Huner NPA, Maxwell DP, Gray GR, Savitch LV, Krol M, et al (1996) Sensing environmental temperature change through imbalances between energy supply and energy consumption: redox state of photosystem II. Physiologia Plantarum 98: 358–364.

[65] Adams WW, Zarter CR, Ebbert V, Demmig-Adams B (2004) Photoprotective strategies of overwintering evergreens. Bioscience 54: 41–49.

[66] Franklin L, Forster R (1997) The changing irradiance environment: consequences for marine macrophyte physiology, productivity and ecology. European Journal of Phycology 32: 207–232.

[67] Nymark M, Valle KC, Brembu T, Hancke K, Winge P, et al. (2009) An integrated analysis of molecular acclimation to high light in the marine diatom *Phaeodactylum tricornutum*. Plos One 4. 10.1371/journal.pone.0007743 PMC2766053 19888450

[68] Takahashi S, Badger MR (2011) Photoprotection in plants: a new light on photosystem II damage. Trends in Plant Science 16: 53–60. 21050798 10.1016/j.tplants.2010.10.001

[69] Weis E (1981) Reversible heat-inactivation of the Calvin cycle: a possible mechanism of the temperature regulation of photosynthesis. Planta 151: 33–39. 24301667 10.1007/BF00384234

[70] Nagao M, Matsui K, Uemura M (2008) *Klebsormidium flaccidum*, a charophycean green alga, exhibits cold acclimation that is closely associated with compatible solute accumulation and ultrastructural changes. Plant Cell and Environment 31: 872–885. 10.1111/j.1365-3040.2008.01804.x 18315534

[71] Nagahisa E, Kanno N, Sato M, Sato Y (1995) Occurrence of free D-alanine in marine macroalgae. Bioscience Biotechnology and Biochemistry 59: 2176–2177.

[72] Gravot A, Dittami SM, Rousvoal S, Lugan R, Eggert A, et al (2010) Diurnal oscillations of metabolite abundances and gene analysis provide new insights into central metabolic processes of the brown alga *Ectocarpus siliculosus* . New Phytologist 188: 98–110. 20862781 10.1111/j.1469-8137.2010.03400.x

[73] Noji M, Inoue K, Kimura N, Gouda A, Saito K (1998) Isoform-dependent differences in feedback regulation and subcellular localization of serine acetyltransferase involved in cysteine biosynthesis from *Arabidopsis thaliana* . Journal of Biological Chemistry 273: 32739–32745. 9830017 10.1074/jbc.273.49.32739

[74] Noctor G, Arisi ACM, Jouanin L, Valadier MH, Roux Y, et al (1997) The role of glycine in determining the rate of glutathione synthesis in poplar. Possible implications for glutathione production during stress. Physiologia Plantarum 100: 255–263.

[75] Noctor G, Arisi ACM, Jouanin L, Foyer CH (1998) Manipulation of glutathione and amino acid biosynthesis in the chloroplast. Plant Physiology 118: 471–482. 9765532 10.1104/pp.118.2.471 PMC34822

[76] López-Maury L, Marguerat S, Bahler J (2008) Tuning gene expression to changing environments: from rapid responses to evolutionary adaptation. Nature Reviews Genetics 9: 583–593. 10.1038/nrg2398 18591982

[77] Collén J, Herve C, Guisle-Marsollier I, Leger JJ, Boyen C (2006) Expression profiling of *Chondrus crispus* (Rhodophyta) after exposure to methyl jasmonate. Journal of Experimental Botany 57: 3869–3881. 17043086 10.1093/jxb/erl171

[78] Sakamoto T, Kamiya T, Sako K, Yamaguchi J, Yamagami M, et al (2011) *Arabidopsis thaliana* 26S proteasome subunits RPT2a and RPT5a are crucial for zinc deficiency-tolerance. Bioscience Biotechnology and Biochemistry 75: 561–567. 10.1271/bbb.100794 21389614

[79] Tanaka K, Tsurumi C (1997) The 26S proteasome: subunits and functions. Molecular Biology Reports 24: 3–11. 9228274 10.1023/a:1006876904158

[80] Coux O, Tanaka K, Goldberg AL (1996) Structure and functions of the 20S and 26S proteasomes. Annual Review of Biochemistry 65: 801–847. 10.1146/annurev.bi.65.070196.004101 8811196

[81] Tanahashi N, Kawahara H, Murakami Y, Tanaka K (1999) The proteasome-dependent proteolytic system. Molecular Biology Reports 26: 3–9. 10363639 10.1023/a:1006909522731

[82] Vallés D, Bruno M, Lopez LMI, Caffini NO, Cantera AMB (2008) Granulosain I, a cysteine protease isolated from ripe fruits of *Solanum granuloso-leprosum* (Solanaceae). Protein Journal 27: 267–275. 18478320 10.1007/s10930-008-9133-4

[83] Grudkowska M, Zagdańska B (2004) Multifunctional role of plant cysteine proteinases. Acta Biochimica Polonica 51: 609–624. 15448724

[84] Vardi A, Berman-Frank I, Rozenberg T, Hadas O, Kaplan A, et al (1999) Programmed cell death of the dinoflagellate *Peridinium gatunense* is mediated by CO2 limitation and oxidative stress. Current Biology 9: 1061–1064. 10508616 10.1016/s0960-9822(99)80459-x

[85] Segovia M, Haramaty L, Berges JA, Falkowski PG (2003) Cell death in the unicellular chlorophyte *Dunaliella tertiolecta*. A hypothesis on the evolution of apoptosis in higher plants and metazoans. Plant Physiology 132: 99–105. 12746516 10.1104/pp.102.017129 PMC166956

[86] Moharikar S, D'Souza JS, Kulkarni AB, Rao BJ (2006) Apoptotic-like cell death pathway is induced in unicellular chlorophyte *Chlamydomonas reinhardtii* (Chlorophyceae) cells following UV irradiation: detection and functional analyses. Journal of Phycology 42: 423–433.

[87] Chaves I, Alves M, Carrilho D, Duque-Magalhaes MC, Ricardo CP, et al (2011) Protein changes during programmed cell death in tobacco. Biologia Plantarum 55: 153–158.

[88] Collén J, Davison IR (1999) Stress tolerance and reactive oxygen metabolism in the intertidal red seaweeds *Mastocarpus stellatus* and *Chondrus crispus* . Plant Cell and Environment 22: 1143–1151.

[89] Collén J, Davison IR (1999) Reactive oxygen production and damage in intertidal *Fucus spp.* (Phaeophyceae). Journal of Phycology 35: 54–61.

[90] Aguilera J, Dummermuth A, Karsten U, Schriek R, Wiencke C (2002) Enzymatic defences against photooxidative stress induced by ultraviolet radiation in Arctic marine macroalgae. Polar Biology 25: 432–441.

[91] Aguilera J, Bischof K, Karsten U, Hanelt D, Wiencke C (2002) Seasonal variation in ecophysiological patterns in macroalgae from an Arctic fjord. II. Pigment accumulation and biochemical defence systems against high light stress. Marine Biology 140: 1087–1095.

[92] Wolfe-Simon F, Grzebyk D, Schofield O, Falkowski P (2005) The role and evolution of superoxide dismutases in algae. Journal of Phycology 41: 453–465.

[93] Bischof K, Rautenberger R, Brey L, Perez-Llorens JL (2006) Physiological acclimation to gradients of solar irradiance within mats of the filamentous green macroalga *Chaetomorpha linum* from southern Spain. Marine Ecology-Progress Series 306: 165–175.

[94] Lu IF, Sung MS, Lee TM (2006) Salinity stress and hydrogen peroxide regulation of antioxidant defense system in *Ulva fasciata* . Marine Biology 150: 1–15.

[95] Burritt D, Larkindale J, Hurd C (2002) Antioxidant metabolism in the intertidal red seaweed *Stictosiphonia arbuscula* following desiccation. Planta 215: 829–838. 12244449 10.1007/s00425-002-0805-6

[96] Luikenhuis S, Perrone G, Dawes IW, Grant CM (1998) The yeast *Saccharomyces cerevisiae* contains two glutaredoxin genes that are required for protection against reactive oxygen species. Molecular Biology of the Cell 9: 1081–1091. 9571241 10.1091/mbc.9.5.1081 PMC25331

[97] Cheng NH, Liu JZ, Brock A, Nelsono RS, Hirschi KD (2006) AtGRXcp, an *Arabidopsis* chloroplastic glutaredoxin, is critical for protection against protein oxidative damage. Journal of Biological Chemistry 281: 26280–26288. 16829529 10.1074/jbc.M601354200

[98] Sundaram S, Rathinasabapathi B (2010) Transgenic expression of fern *Pteris vittata* glutaredoxin PvGrx5 in *Arabidopsis thaliana* increases plant tolerance to high temperature stress and reduces oxidative damage to proteins. Planta 231: 361–369. 19936779 10.1007/s00425-009-1055-7

[99] Grant CM, Luikenhuis S, Beckhouse A, Soderbergh M, Dawes IW (2000) Differential regulation of glutaredoxin gene expression in response to stress conditions in the yeast *Saccharomyces cerevisiae* . Biochimica et Biophysica Acta-Gene Structure and Expression 1490: 33–42. 10.1016/s0167-4781(99)00234-1 10786615

[100] Rouhier N, Gelhaye E, Jacquot J-P (2004) Plant glutaredoxins: still mysterious reducing systems. Cellular and Molecular Life Sciences 61: 1266–1277. 15170506 10.1007/s00018-004-3410-y PMC11138834

[101] Rouhier N, Couturier J, Jacquot J-P (2006) Genome-wide analysis of plant glutaredoxin systems. Journal of Experimental Botany 57: 1685–1696. 16720602 10.1093/jxb/erl001

[102] Collén J, Davison IR (2001) Seasonality and thermal acclimation of reactive oxygen metabolism in *Fucus vesiculosus* (Phaeophyceae) Journal of Phycology. 37: 474–481.

[103] Jaquot JP, Eklund H, Rouhier N, Schürmann P (2009) Structural and evolutionary aspects of thioredoxin reductases in photosynthetic organisms. Trends in Plant Science 14: 336–343. 19446492 10.1016/j.tplants.2009.03.005

[104] Tan S, Greetham D, Raeth S, Grant C, Dawes I, et al (2010) The thioredoxin-thioredoxin reductase system can function in vivo as an alternative system to reduce oxidized glutathione in *Saccharomyces cerevisiae* . Journal of Biological Chemistry 285: 6118–6126. 19951944 10.1074/jbc.M109.062844 PMC2825406

[105] Lemaire SD, Michelet L, Zaffagnini M, Massot V, Issakidis-Bourguet E (2007) Thioredoxins in chloroplasts. Curr Genet 51: 343–365. 17431629 10.1007/s00294-007-0128-z

[106] Vieira Dos Santosa C, Rey P (2006) Plant thioredoxins are key actors in the oxidative stress response. Trends in Plant Science 11: 329–334. 16782394 10.1016/j.tplants.2006.05.005

[107] Arner E, Holmgren A (2000) Physiological functions of thioredoxin and thioredoxin reductase. European Journal of Biochemistry 267: 6102–6109. 11012661 10.1046/j.1432-1327.2000.01701.x

[108] Michelet L, Zaffagnini M, Massot V, Keryer E, Vanacker H, et al (2006) Thioredoxins, glutaredoxins, and glutathionylation: new crosstalks to explore. Photosynthesis Research 89: 225–245. 17089213 10.1007/s11120-006-9096-2

[109] Parsell DA, Lindquist S (1993) The function of heat-shock proteins in stress tolerance: degradation and reactivation of damaged proteins. Annual Review of Genetics 27: 437–496. 10.1146/annurev.ge.27.120193.002253 8122909

[110] Wang W, Vinocur B, Shoseyov O, Altman A (2004) Role of plant heat-shock proteins and molecular chaperones in the abiotic stress response. Trends in Plant Science 9: 244–252. 15130550 10.1016/j.tplants.2004.03.006

[111] Fu WD, Yao JT, Wang XL, Liu FL, Fu G, et al (2009) Molecular cloning and expression analysis of a cytosolic Hsp70 gene from *Laminaria japonica* (Laminariaceae, Phaeophyta). Marine Biotechnology 11: 738–747. 19259734 10.1007/s10126-009-9188-z

[112] Vayda ME, Yuan ML (1994) The heat-shock response of an Antarctic alga is evident at 5-degrees-C. Plant Molecular Biology 24: 229–233. 8111021 10.1007/BF00040590

[113] Fu W, Shuai L, Yao J, Zheng B, Zhong M, et al (2011) Molecular cloning and expression analysis of a cytosolic Hsp70 gene from *Ulva pertusa* (Ulvophyceae, Chlorophyta). Journal of Applied Phycology 23: 681–690.

[114] Li R, Brawley SH (2004) Improved survival under heat stress in intertidal embryos (*Fucus spp.*) simultaneously exposed to hypersalinity and the effect of parental thermal history. Marine Biology 144: 205–213.

[115] Pearson G, Hoarau G, Lago-Leston A, Coyer J, Kube M, et al (2010) An expressed sequence tag analysis of the intertidal brown seaweeds *Fucus serratus* (L.) and *F. vesiculosus* (L.) (Heterokontophyta, Phaeophyceae) in response to abiotic stressors. Marine Biotechnology 12: 195–213. 19609612 10.1007/s10126-009-9208-z

[116] Wernberg T, Thomsen MS, Tuya F, Kendrick GA, Staehr PA, et al (2010) Decreasing resilience of kelp beds along a latitudinal temperature gradient: potential implications for a warmer future. Ecology Letters 13: 685–694. 20412279 10.1111/j.1461-0248.2010.01466.x

[117] Alexieva V, Ivanov S, Sergiev I, Karanov E (2003) Interaction between stresses. Bulg J Plant Physiol Special Issue: 1–17.

[118] Müller R, Laepple T, Bartsch I, Wiencke C (2009) Impact of oceanic warming on the distribution of seaweeds in polar and cold-temperate waters. Botanica Marina 52: 617–638.

[119] Starr RC, Zeikus JA (1993) Utex – the culture collection of algae at the university of Texas at Austin 1993 list of cultures. Journal of Phycology 29: 1–106.

[120] Heinrich S, Frickenhaus S, Glöckner G, Valentin K (2012) A comprehensive cDNA library of temperature stressed *Saccharina latissima* (Phaeophyceae). European Journal of Phycology 47: 83–94.

[121] Conesa A, Götz S, Garcia-Gomez J, Terol J, Talon M, et al (2005) Blast2GO: a universal tool for annotation, visualization and analysis in functional genomics research. Bioinformatics 21: 3674–3676. 16081474 10.1093/bioinformatics/bti610

[122] Powell S, Szklarczyk D, Trachana K, A R, Kuhn M, et al (2012) eggNOG v3.0: orthologous groups covering 1133 organisms at 41 different taxonomic ranges. Nucleic Acids Research 40 (D1): 284–289. 10.1093/nar/gkr1060 PMC3245133 22096231

[123] Altschul SF, Madden TL, Schäffer AA, Zhang J, Zhang Z, et al (1997) Gapped BLAST and PSI-BLAST: a new generation of protein database search programs. Nucleic Acids Research 25: 3389–3402. 9254694 10.1093/nar/25.17.3389 PMC146917

